# Health Safety Assessment of Ready-to-Eat Products Consumed by Children Aged 0.5–3 Years on the Polish Market

**DOI:** 10.3390/nu14112325

**Published:** 2022-06-01

**Authors:** Anita Żmudzińska, Anna Puścion-Jakubik, Joanna Bielecka, Monika Grabia, Jolanta Soroczyńska, Konrad Mielcarek, Katarzyna Socha

**Affiliations:** Department of Bromatology, Faculty of Pharmacy with the Division of Laboratory Medicine, Medical University of Białystok, Mickiewicza 2D Street, 15-222 Białystok, Poland; anna.puscion-jakubik@umb.edu.pl (A.P.-J.); joanna.bielecka@umb.edu.pl (J.B.); monika.grabia@umb.edu.pl (M.G.); jolanta.soroczynska@umb.edu.pl (J.S.); konrad.mielcarek@umb.edu.pl (K.M.); katarzyna.socha@umb.edu.pl (K.S.)

**Keywords:** baby food, food contaminant, toxic elements, food exposure, children safety, children’s health, arsenic, cadmium, mercury, lead

## Abstract

Toxic elements have a negative impact on health, especially among infants and young children. Even low levels of exposure can impair the normal growth and development of children. In young children, all organs and metabolic processes are insufficiently developed, making them particularly vulnerable to the effects of toxic elements. The aim of this study is to estimate the concentration of toxic elements in products consumed by infants and young children. The health risk of young children due to consumption of ready-made products potentially contaminated with As (arsenic), Cd (cadmium), Hg (mercury), and Pb (lead) was also assessed. A total of 397 samples (dinners, porridges, mousses, snacks “for the handle”, baby drinks, dairy) were analyzed for the content of toxic elements. Inductively coupled plasma mass spectrometry (ICP-MS) was used to assess As, Cd, and Pb concentration. The determination of Hg was performed by atomic absorption spectrometry (AAS). In order to estimate children’s exposure to toxic elements, the content of indicators was also assessed: estimated daily intake (EDI), estimated weekly intake (EWI), provisional tolerable weekly intake (PTWI), provisional tolerable monthly intake (PTMI), the benchmark dose lower confidence limit (BMDL), target hazard quotient (THQ), hazard index (HI), and cancer risk (CR). The average content of As, Cd, Hg, and Pb for all ready-made products for children is: 1.411 ± 0.248 µg/kg, 2.077 ± 0.154 µg/kg, 3.161 ± 0.159 µg/kg, and 9.265 ± 0.443 µg/kg, respectively. The highest content As was found in wafer/crisps (84.71 µg/kg); in the case of Cd, dinners with fish (20.15 µg/kg); for Hg, dinners with poultry (37.25 µg/kg); and for Pb, fruit mousse (138.99 µg/kg). The results showed that 4.53% of the samples attempted to exceed Pb, and 1.5% exceeded levels of Hg. The highest value of THQ was made in the case of drinks, for Cd and Pb in mousses for children, and Hg for dairy products. The THQ, BMDL, and PTWI ratios were not exceeded. The analyzed ready-to-eat products for children aged 0.5–3 years may contain toxic elements, but most of them appear to be harmless to health.

## 1. Introduction

Toxic elements are found in the human environment, and, as such, they can have a negative effect on health. Their consumption is especially dangerous in the early years of life, as this can affect children’s development, even at low exposure levels. Among infants and young children, changes in the structure and function of the main organs occur very quickly, making them a group more susceptible to the negative effects of toxic elements [1]. Infants and young children are characterized by a higher resting metabolism and a higher food consumption per kilogram of body weight. Additionally, changes in body composition can affect the absorption, distribution, and storage of toxic elements in the organs of a growing organism. The digestive and endocrine systems are not fully developed at the time of birth, and vary in their maturation rates. Renal filtration among young children is only 30–40% developed. The differences in the development of the organism affect the degree of exposure and the distribution of pollutants; thus, the effects of exposure may be much more severe than in adults [1].

The inorganic form of arsenic (As) is listed by the IARC (Group 1) as a carcinogen [2]. Exposure to As has a toxic effect on diseases of the nervous system, children’s nervous development, respiratory system, and skin. The typical sources of As in food are rice processing products, dairy products, and products intended for infants [3].

Cadmium (Cd) is also on the IARC list of carcinogenic pollutants (Group 1) [2]. As with Pb, Cd can be neurotoxic in children at doses lower than the TWI (tolerable weekly intake). Exposure to Cd disrupts osteoblast metabolism, collagen production, and also increases the urinary excretion of calcium and phosphorus. In children, it can disturb growth and damage bones [4]. Cd is present in grains, rice, fish and seafood, and vegetables [5].

Mercury (Hg) is not classified in the IARC list for carcinogenicity (Group 3) [2]. Hg enters the body through food, contaminated water, cosmetics, soil, and other Hg-containing products. The toxicity of this element depends on the time of exposure, dose, and type of Hg compound. Methylmercury (MeHg) is a carbon-bound form of Hg that is highly neurotoxic in infants and young children [6]. MeHg may cause central nervous system dysfunction in children. Exposure to Hg can cause neuromotor dysfunction, mental retardation, and cerebral palsy [7]. In adults, the half-life of Hg can be up to 90 days, and in children this time may be prolonged [8]. The main source of Hg is fish and seafood, with the highest amounts being among predatory fish [6].

Lead (Pb) is recognized as “possibly carcinogenic to humans (Group 2B)” in the International Agency of Research on Cancer (IARC) list of carcinogenic contaminants [2]. Its neurotoxic effect was observed at the lowest tested level, therefore a safe threshold for exposure to Pb cannot be assumed [9]. Exposure to Pb can impair the development of young children by damaging the nervous system. Long-term exposure results in difficulties with concentration and attention. and a lower IQ [10]. The higher the Pb concentration in the blood, the more nervous system disorders develop in children [11]. Pb can be found in meat, fish and seafood, grain products, dairy products, and fruit and vegetables [12].

In the European Union (EU), products for infants and young children are subject to specific standards regarding the composition, nutritional value, and contamination of food. The regulation requires food producers to evaluate toxic elements in products intended for infants and young children. According to the regulation of the European Commission, the permissible concentrations of toxic elements in children’s products are more restrictive compared to conventional food [13].

In Poland and in Europe, there are no extensive publications assessing the content of toxic elements in children’s products. Studies from outside of Europe show that the content of As and Cd are exceeded in 75% and 14% of samples tested; therefore, there is a need to evaluate food for this age group [14,15].

The aim of the study was to assess the safety of ready-to-eat products for children aged 0.5–3 years in terms of the content of toxic elements. In addition, the health risk of infants and young children as a result of exposure to toxic elements in ready-to-eat food was assessed and compared with the applicable standards.

## 2. Materials and Methods

### 2.1. Sample Collection

The research material consisted of 397 samples. Table 1 shows the number of samples with product categories.

The aim was to collect as many different categories as possible to obtain representative groups. The selection of samples reflects the range of products on the Polish market. The samples were purchased in brick-and-mortar stores (hypermarkets, discount stores, and children’s food stores located in Białystok) in north-eastern Poland, as well as in Polish online stores between December 2020 and September 2021. We have selected the most accessible and most consumed products by children aged 0.5–3 years old. The products were sourced from leading baby food producers, such as Nestle, Nutricia, Hipp, Humana, Holle, and Helpa. Most of the analyzed products can be consumed by children from 6 months of age, while products such as biscuits, cookies, crips and fruit bars should be consumed after 12 months of age. Conventional products for the majority of consumers were also analyzed, such as cheese, milk desserts, and yoghurts, since they are often eaten by children.

### 2.2. Sample Digestion

All products were homogenized in a grinder or ground in a mortar, weighed (sample weight 0.25–0.35 g, with accuracy 0.001 g), and then placed in mineralization vessels made of polytetrafluoroethylene. The next step was the addition of 4 mL of concentrated 69% HNO_3_ (Berghof, Speedwave, Eningen, Germany). The reagent was added according to the recommendations of the producer. Closed-loop microwave mineralization took place in four phases: first phase with a duration of 10 min, at a temperature of 170 °C, pressure 20 atm, power 90%; second phase with a duration of 10 min, at a temperature of 190 °C, pressure 30 atm, power 90%; third phase with a duration of 40 min, temperature 210 °C, pressure 40 atm, power 90%; and fourth phase with a cooling duration of 18 min, temperature 50 °C, pressure 40 atm, power 0%. After mineralization, the samples were quantitatively transferred to polypropylene vessels and diluted 10 times.

### 2.3. Analysis of Toxic Elements

In accordance with the regulations of the European Commission [16,17], individual types of food products have a daily limit of the consumption of toxic elements, which is the level at which they are safe for health.

#### 2.3.1. As, Cd, Pb

Inductively coupled plasma mass spectrometry (ICP-MS NexION 300D, PerkinElmer, Waltham, MA, USA) with a kinetic discrimination chamber (KED) was used for As analysis, while Cd and Pb analysis was performed in standard mode. To correct for polyatomic interference in this configuration, kinetic energy discrimination and collisions were applied. The limit of detection (LOD) was determined by producing 10 independent blank measurements. The LOD values were as follows: for As: 0.019 µg/kg; for Cd: 0.017 µg/kg; for Pb: 0.16 µg/kg, taking LOD as a three-fold standard deviation from the mean value of the sample concentration.

#### 2.3.2. Hg

Hg analysis was determined without the initial mineralization process. Determination of Hg was performed by atomic absorption spectrometry (AAS) using the amalgamation technique (AMA-254, Leco Corp., Altec Ltd., Prague, Czech Republic). The samples were weighed (sample weight 0.018–0.023 g), placed in a nickel cuvette, and analyzed. In the first step, the sample was dried and ashed with oxygen at 600 °C. Hg vapors were collected by the amalgamator. In the last step, the element was released from the amalgamator and measured by atomic absorption spectrometry at 254 nm. The LOD for each sample was 0.003 ng/kg.

#### 2.3.3. Certified Reference Materials

Before starting the analyses, the certified reference material (CRM) was marked to verify the accuracy of the method. In the case of baby dinners, it was Simulated diet D (Swedish National Food Administration, Livsmedelsverket, Uppsala, Sweden); for porridges and snacks—corn flour (Institute of Nuclear Chemistry and Technology, Warsaw, Poland); for dairy products—skim milk powder (Community Bureau of Reference BCR). A total of six independent samples were performed for each CRM. Table 2 shows the results of the quality control of the certified reference materials.

### 2.4. Risk Assessment

To estimate the exposure to toxic elements in a short time, indicators such as estimated daily intake (EDI), estimated weekly intake (EWI), provisional tolerable weekly intake (PTWI), provisional tolerable monthly intake (PTMI) were used. Long-term exposure to toxic elements was estimated using the following indicators: the benchmark dose lower confidence limit (BMDL), target hazard quotient (THQ), hazard index (HI), and cancer risk (CR) [18].

Currently, the application of the PTWI index for Pb and As, previously recommended by EFSA in 2002, has been withdrawn. A more appropriate indicator is the BMDL, which is used to compare the coverage of the benchmark dose lower confidence limit [18].

In order to determine the dose of the oral exposure of toxic elements, the EDI values were calculated in accordance with the equation:

EDI = C × Cons,

where C is the average concentration of a given toxic element in products for children (mg/kg), and Cons is the average daily consumption (kg) of product (children’s dinners, porridges, mousses, drinks, snacks, dairy products) by young children aged 0.5–3 years (average: 1 year and 9 months) in Poland [19]. Standard portions consumed by children and declared by the producer were adopted. Based on the EDI value, the average weekly consumption (EWI) was calculated by multiplying the given result by 7 (7 days).

The PTWI value was also calculated according to the equation:

PTWI = EDI × 7/BW,

where BW is the average body weight (the adopted average body weight for this age group is 10.5 kg). The PTWI value for Cd is 7 µg/kg BW/day, and for Hg, it is 1.6 µg/kg BW/day [16,20].

The BMDL index was also determined according to the equation:

BMDL = EDI/BW

BMDL values for As are 3 µg/kg/BW/day, with a benchmark dose lower confidence of 0.5% (BMDL0.5) according to FAO/WHO standards. For Pb, it is 0.5–6 µg/kg BW/day. PTWI values which are above the levels set by the standards may pose a health hazard. The results are presented as a percentage of the reference value [18].

The THQ index is also determined to produce adverse health effects due to the properties of the toxic elements, according to the equation:

THQ = (Fr × D × Cons × C)/(RfD × BW × T) × 10^−3^,

where Fr is the frequency of exposure per year (365 days), D is the exposure time (70 years), Cons is the average daily consumption of the product [g], C is the average concentration of a given toxic element in the product, and RfD is the oral reference dose, which is respectively 0.3 µg/kg BW/day for As and Hg, and 1 µg/kg BW/day for Cd and Pb, according to the guidelines of the United States Environmental Protection Agency (US EPA) [17]. If the THQ value is >1, it indicates a potential non-carcinogenic risk; if THQ is <1, it indicates a low non-carcinogenic risk [21].

Cancer risk (CR) is used to determine the carcinogenic risk for each toxic square, calculated according to the equation:

CR = (Fr × D × EDI × SF)/T × 10^−3^,

where SF stands for the slope factor of cancer. According to US EPA standards, the SF value is 1.5 mg/kg/day for As, 6.3 mg/kg/day for Cd, and 0.0085 mg/kg/day for Pb. If the CR index is above 10^−4^, it refers to an increased carcinogenic risk. The THQ and CR values for Hg were not calculated since, according to the US EPA position, it is not considered a carcinogenic element [21].

### 2.5. Statistical Analysis

The obtained results were analyzed using the Statistica software (TIBCO Software Inc., Palo Alto, CA, USA). The Shapiro–Wilk test was performed, and the distribution was found to be non-normal. Non-parametric Mann–Whitney U tests and the Kruskal–Wallis ANOVA test were used to analyze the concentrations of toxic elements in various product groups. The results were summarized using the median and quartiles. To compare the results of our own research with other authors, the tables contain the mean, along with the standard deviation, as well as the maximum and minimum values. In order to assess significance, the values *p* < 0.05, *p* < 0.01, and *p* < 0.001 were adopted.

## 3. Results

### 3.1. Content of As, Cd, Hg, and Pb

**Figure 1:**
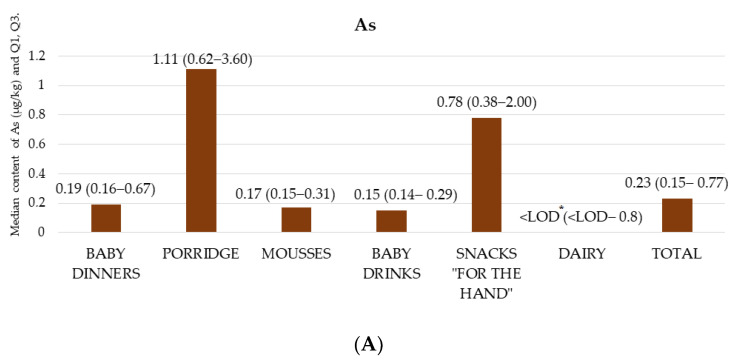

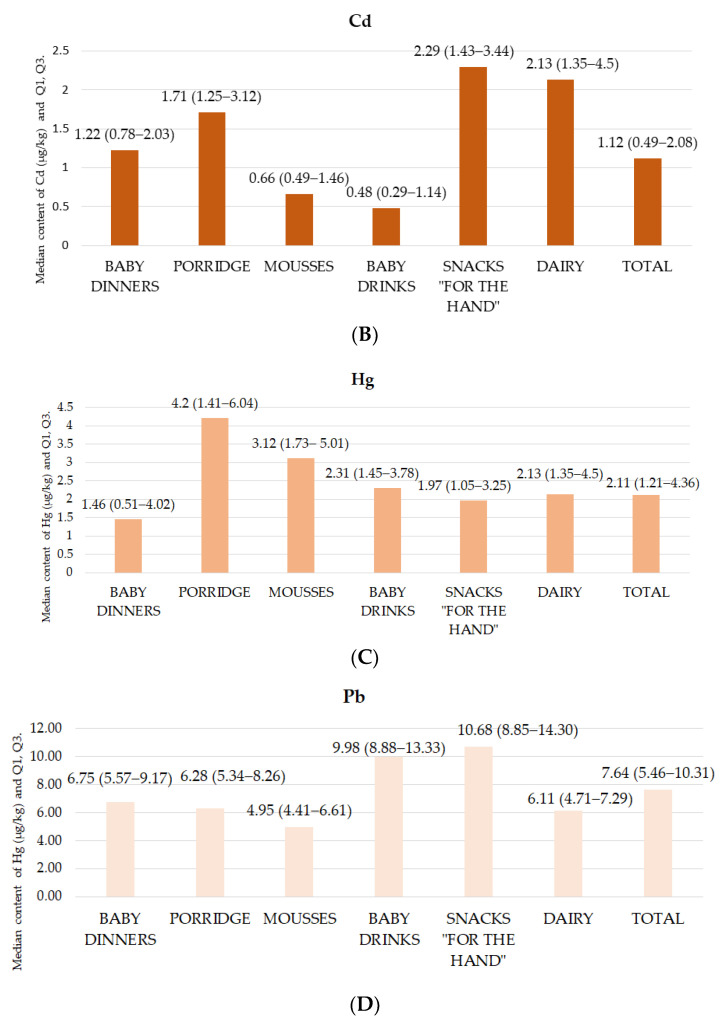
Median As (**A**), Cd (**B**), Hg (**C**) and Pb (**D**) content with quartile 1 (Q1) and quartile 3 (Q3) in the analyzed product groups. * LOD—limit of detection.

The average content of As in all samples was 1.41 ± 0.25 µg/kg, while in 69 samples, no As was recorded. These were mainly products without the addition of cereals (fruit and vegetable mousses, drinks and dairy products), and the highest value was determined in a fruit bar with the addition of rice (84.71 µg/kg). Among all of the categories, the lowest As content was found in fruit and cereal mousses (0.16 ± 0.09 µg/kg) and dairy products (0.10 ± 0.16 µg/kg). In turn, the highest As value was recorded in porridges (2.30 ± 0.33 µg/kg), and, in particular, in gluten-free porridges (4.31 ± 0.72 µg/kg). High concentrations were also obtained in snacks “for the hand” (2.92 ± 10.77 µg/kg), and especially in wafers/chips (4.88 ± 15.34 µg/kg). In accordance with the level of the European Commission [22], the maximum allowable level of As in food is 100 µg/kg (in cereals, fruit and vegetables, and meat). There were 69 samples for As below the limit of detection. The median As content results with quartile 1 (Q1) and quartile 3 (Q3) in the analyzed product groups are presented in Figure 1A.

The mean concentration of Cd in all the tested samples was 2.08 ± 0.15 µg/kg. The lowest concentration of Cd in products was 0.14 µg/kg, recorded in juices for children (apple, grape, chokeberry, raspberry). The highest Cd concentration was 20.15 µg/kg, and was in a ready-to-eat dinner for children after 6 months of age, consisting mainly of vegetables and salmon. Taking into account the product categories, the lowest Cd values were recorded in dairy products (0.95 ± 2.56 µg/kg) and especially in the yoghurt subgroup (0.45 ± 0.78 µg/kg), mousses (1.39 ± 2.31 µg/kg), and also in fruit juices (0.66 ± 2.01 µg/kg). The highest levels of Cd were obtained in drinks for children (3.39 ± 1.30 µg/kg), especially in fruit drinks and water (4.17 ± 5.98 µg/kg) and snacks “for the hand” (3.09 ± 1.69 µg/kg), especially in the waffle/crisps subcategory (3.51 ± 3.38 µg/kg). According to the European Commission Regulation (EU) 2021/1323 of 10 August 2021 amending Regulation (EC) No 1881/2006 as regards maximum levels of Cd in certain foodstuffs, the maximum level of Cd is 100 µg/kg for cereal, 40 µg/kg for baby food, 20 µg/kg for fruits and vegetables, 20 µg/kg for juices, and 50 µg/kg for meat and fish [17]. The daily limit for Cd was not exceeded; therefore, it has not been included in the Table 3. There were 47 samples for Cd below the limit of detection. The median Cd content results with quartile 1 (Q1) and quartile 3 (Q3) in the analyzed product groups are presented in Figure 1B.

The average Hg level in all samples was 3.16 ± 0.16 µg/kg, The lowest value was recorded in the fruit and cereal bar (the main ingredients of which are grape juice, oatmeal, apple juice) at 0.005 µg/kg, while the highest Hg value was 37.25 µg/kg in a lunch based on vegetables, rice, and hake. Among all categories, the lowest amounts of Hg were found in snacks “for the hand” (2.36 ± 1.69 µg/kg) and in the subcategory of vegetable mousses (2.26 ± 0.83 µg/kg). The highest amounts of Hg were recorded in porridges (4.20 ± 0.35 µg/kg), in particular in gluten-free (4.89 ± 0.69 µg/kg) and gluten (4.86 ± 0.60 µg/kg) porridges; in the subcategories, the highest levels were recorded in fish dinners (as much as 9.213 ± 0.571 µg/kg) and fruit and vegetable mousses (5.748 ± 1.118 µg/kg). In accordance with European Commission Regulation (EU) 2018/73 of 16 January 2018 amending Annexes II and III to Regulation (EC) No 396/2005 of the European Parliament and of the Council, regarding the maximum residue levels for Hg compounds in or on certain products, the maximum allowable level of Hg is: 5 µg/kg for fish, 10 µg/kg for cereal, baby food, fruit and vegetables, meat, and milk, and 20 µg/kg for juices [16]. Table 3 shows the number of samples that exceeded the maximum allowable limits of Hg and Pb. The limit for Hg was exceeded in 6 samples, which constitutes 1.5% of all samples. The highest amount of the exceeded Hg limit was recorded in lunches (three samples). The daily limit was not exceeded in porridges, snacks for children, and dairy products. There were the same samples for Hg below the limit of detection. The median Hg content results with quartile 1 (Q1) and quartile 3 (Q3) in the analyzed product groups are presented in Figure 1C.

The mean concentration of Pb in all samples was 9.27 ± 0.44 µg/kg, with the lowest recorded concentration being 0.46 µg/kg in children’s juice (based on apple, grape, aronia, raspberry). The highest Pb value was 138.99 µg/kg, found in a mousse (based on apple, cottage cheese, and grape juice). Among all categories, the lowest Pb level was recorded in drinks for children (1.14 ± 0.98 µg/kg). The highest concentrations of Pb were found in snacks “for the hand” (12.80 ± 7.56 µg/kg), porridges (8.09 ± 0.70 µg/kg), mousses for children (7.97 ± 0.02 µg/kg), especially fruit-based mousses (9.40 ± 4.06 µg/kg), and in children’s dinners (7.55 ± 0.41 µg/kg). According to the regulations of the European Commission, the maximum permissible level of Pb is: 10 µg/kg for meat, 20 µg/kg for cereal, baby food and milk, 30 µg/kg for fish, 50 µg/kg for juices, and 100 µg/kg for fruit and vegetables [20]. The median Pb content results with quartile 1 (Q1) and quartile 3 (Q3) in the analyzed product groups are presented in Figure 1D. There were 10 samples for Pb below the limit of detection.

Detailed content of toxic elements in individual groups and subgroups is presented in Table S1 (Supplementary Materials).

There were 18 exceedances in the content of the daily Pb limit (4.53%). The highest Pb content was recorded in drinks (seven samples) and baby snacks (six samples). The lowest Pb exceedances were recorded in dairy products (one sample), mousses (one sample), and dinners (one sample).

Table 4 presents statistically significant differences in the content of toxic elements in the analyzed products. The most statistically significant differences were noted in the As content. The As content in lunches differed statistically significantly in drinks (*p* < 0.01). Differences were also noted in porridges and drinks (*p* < 0.01), mousses and drinks (*p* < 0.01), baby snacks and drinks (*p* < 0.01), dairy and drinks (*p* < 0.01), and groups: poultry dinners and fish dinners (*p* < 0.001), beef dinners and fish dinners (*p* < 0.001), beef dinners and fish dinners (*p* < 0.001), pork dinners and fish dinners (*p* < 0.001), rabbit dinners and fish dinners *(p* < 0.001), vegetarian dinners and fish dinners (*p* < 0.001), and milk porridge and gluten-free cereal (*p* < 0.001).

There were also statistically significant differences in the Hg content in mousses and dairy (*p* < 0.05), as well as in the pork dinners and vegetarian dinners subgroup (*p* < 0.05). For the Pb content, one difference was found between fruit drinks and water–fruit juices (*p* < 0.01). In this study, no statistically significant differences in the Cd content were found.

### 3.2. Risk Assessment

The health indicators of all analyzed products are summarized in Table 5. In the case of PTWI and BMDL indicators, no exceedances were found in the tested assortment.

Baby dinners have the highest EDI and EWI values for As and Cd (EDI_As_ = 0.419 ± 0.909 mg/day, EWI_As_ = 2.938 ± 6.368 mg/week, EDI_Cd_ = 0.515 ± 0.744, EWI_Cd_ = 3.607 ± 5.205). The estimated daily consumption was the lowest in dairy products in the case of As (EDI = 0.007 ± 0.034 mg/day, EWI = 0.054 ± 0.233 mg/week), while in the case of Cd, this was in dairy products (EDI = 0.063 ± 0.081 mg/day, EWI = 0.436 ± 0.563 mg/week) and porridges (EDI = 0.064 ± 0.065 mg/day, EWI = 0.449 ± 0.459 mg/week). Baby dinners showed the highest value of EDI and EWI in the case of Pb (EDI = 1.651 ± 0.922 mg/day, EWI = 11.563 ± 6.458 mg/week), while in the case of Hg, the highest value was found in mousses (EDI = 0.381 ± 0.239 mg/day, EWI = 2.669 ± 1.677 mg/week). The lowest EDI value for Hg and Pb was determined in porridges (EDI_Hg_ = 0.097 ± 0.057 mg/day, EWI_Hg_ = 0.681 ± 0.402 mg/week, EDI_Pb_ = 0.187 ± 0.114 mg/day, EWI_Pb_ = 1.313 ± 0.804 mg/week). In the case of the PTWI and BMDL indicators, the specified limits were not exceeded (Table 5). The PTWI in all ready-to-eat samples for Cd was 0.157 ± 0.308 µg/kg, which was 2.25% of the norm; for Hg, it was 0.221 ± 0.308 µg/kg, which was 13.84% of the norm. The BMDL_As_ was 0.016 ± 0.06 µg/kg, which was 0.54% of the norm, while in the case of Pb, it was 0.101 ± 0.119 µg/kg (20.23% of the norm).

The estimated THQ and CR ratios for the analyzed toxic elements are presented in Table 6. The highest values of the THQ index for all elements were recorded for Hg in dairy products (1.14 × 10^−7^ ± 6.18 × 10^−7^). In the case of As, the highest value of the index was 1.41 × 10^−7^ ± 3.04 × 10^−7^, and these levels were found in baby dinners. In the case of Cd, the highest THQ was recorded in porridges for children, and THQ was 1.31 × 10^−8^ ± 2.01 × 10^−8^. In the case of Hg, it was 1.01 × 10^−7^ ± 1.19 × 10^−7^, found in dinners for children. In the case of Pb, it was porridges 1.01 × 10^−7^ ± 1.19 × 10^−7^. The norms of THQ values were not exceeded in the tested assortment, therefore there is no increased risk of non-carcinogens effect. The CR index was not exceeded, so the research shows that the risk of developing cancer as a result of consuming the analyzed products is low.

The analyzed products were also divided according to the intended use of the given products in terms of age category: for children 6–12 months, for children under 12 months, and products without an age declaration. Table 7 presents the characteristics of age groups in terms of mean, median, and other statistical parameters. The age declaration group includes dinners, porridges, and mousses. The remaining product groups (drinks, snacks, and dairy products for children) are included in the category without an age declaration.

The highest average contents of As, Cd, and Hg were recorded in the group of products intended for children aged 6–12 months (As: 2.01 ± 3.67 µg/kg, Cd: 2.22 ± 3.14 µg/kg, Hg: 3.20 ± 2.86 µg/kg). In the case of Pb, it was in the group of products without age declaration (10.95 ± 7.02 µg/kg). The highest mean content of As was recorded in the group of products for children 6–12 months (2.01 ± 3.67 µg/kg), and the lowest for children under 12 months (1.01 ± 2.54 µg/kg). The highest mean Cd content was observed in the group of products for children aged 6–12 months (2.22 ± 3.14 µg/kg), and the lowest in the group of products without declaration (1.98 ± 3.23 µg/kg), although these differences were small (the statistical significance of the analyzed groups was presented in Table 7). The highest mean Hg content was recorded in the group of products intended for children 6–12 months (3.20 ± 2.86 µg/kg), and the lowest mean in the group of products without declaration (2.68 ± 1.86 µg/kg), while there were not statistically significant groups (Table 7). In the case of Pb, the highest mean was recorded in the group of products without age declaration (10.95 ± 7.02 µg/kg), and in the remaining groups the mean was very similar (in the group of products for children 6–12 months: 7.82 ± 4.76 µg/kg, in the group above 12 months: 7.74 ± 13.55 µg/kg).

The most statistically significant differences were recorded in the case of Pb. The Pb content differed statistically significantly in the group of products intended for children aged 6–12 months and over 12 months (*p* < 0.01); in the group of 6–12 months and in the first group without age declaration (*p* < 0.001); in the group of products above 12 months month and the product group without age declaration (*p* < 0.001). In the case of As, statistical significance was noted in the groups of products for children aged 6–12 months and over 12 months of age (*p* < 0.01); and in the group of 6–12 months in the group without age declaration (*p* < 0.001). The content of Cd differed statistically significantly in the group of products intended for children 6–12 months, the group without age declaration (*p* < 0.001); and in the group over 12 months of age, and the group without age declaration (*p* < 0.01).

## 4. Discussion

In our study, we found that toxic elements are present in ready-to-eat baby foods, and that some foods contain Hg and Pb levels of concern.

Our research found that the permissible daily limit was not exceeded for As. In a Spanish study, As was detected in three samples, one of which exceeded the acceptable toxicological standards [23]. The study by Rothenberg et al., (2017) examined the content of As in rice products for children. These products had a higher content of the tested elements compared to wheat and oat products, but they did not exceed the permissible standard [24]. A similar study by Gu et al., (2020) assessed the As content in products for infants and young children. Despite the fact that total As was found in 88% of the samples, it did not exceed the total permissible content, while 75% of the samples exceeded the standard for inorganic As for infants and young children [14]. In turn, Ljung et al., (2011) assessed the content of toxic elements in, among others, products based on milk, oats, spelt, and whole grain rice. The researcher showed that rice-based products were the largest source of As (up to 30 μg/kg) [25]. A Nigerian study (2020) showed that the tolerable daily intake of As was exceeded in baby food. The mean daily consumption of As was 437.1 µg/kg (771% PTDI). Abnormalities for As have been observed in products containing rice [26]. Rice is the most abundant product containing As. In Poland, rice is consumed rarely, and among children under 3 years of age, the average consumption of rice is 17.9 g/day The range of rice-based products on the Polish market is relatively small, and they are not so widely consumed by children, so should not pose a threat to children’s health.

In our own study, the maximum allowable content was not exceeded for Cd. Gardener et al., (2019) showed that Cd was found in 57% of samples. Out of 91 trials of baby food, 14% exceeded the acceptable limits for Cd consumption [15]. The study by Kim et al., (2014) assessed the exposure to toxic elements in the daily food ration. The mean concentration of Cd in the diet was 0.38 ± 0.20 μg/kg BW/day, respectively. In the case of Cd, the reference value was exceeded (42%) [27]. De Castro et al., (2010) Cd concentrations were higher than in our study, but did not exceed the acceptable limits (109 μg/kg) [28]. In the study (2019), the mean daily exposure to Cd was 0.43 µg/kg BW/day [29]. In our study, the average daily consumption for Cd was lower (0.224 ± 0.439 µg/kg). In the study by Winiarska-Mleczan (2012), the average consumption in one meal (ready-made dessert for children) covers 2% of the PTDI of Cd, while the average consumption of a bottle of drink for children is 2% of the PTDI of Cd [30]. In a study, Chen et al., (2021) showed that, among all age groups, children up to 5 years old showed the highest concentrations of Cd and the highest risk of health risk. The total Cd exposure index for children aged 0.5–5 years was 0.00325, and the Cd level was 3.9 times the PTMI norm on Cd, suggesting that children are at an unacceptable level of health risk. [31]. This is a study carried out in China, where there are different eating habits than in Europe (e.g., higher consumption of e.g., rice), which may be the reason for such a high rate. An Egyptian study analyzed the content of Cd in dairy products consumed by children. The estimated daily intake for Cd was 0.33 μg/kg bw/day, which is 39.8% of the TDI [32]. In comparison, in our study, the EDI for Cd in dairy products was higher and amounts to 0.43 ± 0.56 μg/kg. In our study, the highest Cd value was found for the salmon-based children’s dinner (20.15 µg/kg), which may suggest greater caution in terms of children’s consumption of fish. Among different groups, the highest value of Cd was recorded in drinks for children. The reason for high concentrations of Cd may be the high presence of Cd in the water due to the affinity for its accumulation [12]. The presence of Cd in children’s products should be of concern, since Cd is neurotoxic in amounts lower than the reference doses, which means that any dose may have adverse health effects and impair children’s development.

In our study, the maximum allowable content was exceeded for Hg in 1.5% of samples analyzed. In the study by Guerin et al., (2018), the Hg content of 291 products for infants and young children, as well as conventional foods consumed by this group, on the French market was determined. This element was detected in 7.6% of all samples, while none of the products exceeded the permitted Hg limit [33]. Higher Hg concentrations were also found in rice products, as compared to oat products [24]. In our study, the product with the highest Hg content was based on rice and hake. In the study of Spungen et al., (2019), the mean daily exposure to Hg was 0.12 µg/kg bw/day in children aged 1–3 years old [29]. In our study, the estimated daily intake was higher (0.31 ± 0.44 µg/kg). The study by Martins et al., (2013) assessed the exposure of infants and young children to Hg derived from processed cereal-based foods and infant food (vegetable-, meat-, fish-, and fruit-based meals). It has been shown that the average consumption of the analyzed products is from 0.2 to 0.4% of the PTWI of Hg [34]. In our study, the coverage of PTWI of Hg is significantly higher (9.6%), but it should not be a cause for concern. The study by Igwaze et al., (2020) showed that mean daily consumption of Hg was 23.7 µg/kg (41.8% of PTDI). The mean daily Hg intake was below the PTDI value [26]. In a Spanish study evaluating infant dietary Hg exposure, the EDI for Hg was shown to be significantly higher, at 1.5 µg/kg BW/day (EWI = 11 BW/day). In our study, the EDI for Hg was 0.31 ± 0.43 μg/kg BW/day (EWI = 2.20 ± 0.07 μg/kg) [35]. Moreover, it is particularly interesting for public health that the tested samples showed exceeded Hg concentrations in six samples, including three categorized as children’s lunches, two of which contained fish. This should be of concern, especially in view of the potential risks posed by frequent consumption of fish-based products. Hg damages the central nervous system of children, impairs the immune function, and leads to dysfunction of the children’s bloodstream.

In our study, the maximum allowable content was exceeded for Pb in 4.5% of samples. The Gardener et al., (2019) study assessed the content of Pb in products consumed by infants and young children (infant food, cereals, drinks, pouches). Pb was found in 37% of samples [15]. In the study by Škrbić et al., (2016) which assessed the content of toxic elements in 90 products for children (porridge fruit, porridge vegetable, meat and fish, porridge, corn and rice porridge, and yogurt-based products), Pb was detected in two products [23]. Ljung et al., (2011) showed that Pb was found in rice-based products (13 μg/kg) [25]. In the study by Kim et al., (2014), the mean concentration of Pb was 0.47 ± 0.14 μg/kg BW/day and 35% of samples exceeded the maximum allowable content limit [27]. In a study by Rasic Misic et al., (2022), Pb was detected in 31% of baby and toddler juice and chafing samples. The mean concentrations of Pb were 12.0 μg/kg in fruit-based food, 29.0 μg/kg in vegetable-based food, and 34.50 μg/kg in meat-based food. In all juices, the Pb value was higher than the maximum concentration in food for young children (0.05 mg/kg). In our research, similar conclusions were noted, since the most exceedances of the permissible daily norm were recorded in beverages—1.76% of all products (7 samples). In our study, mean Pb concentrations were also high in fruit-based mousses (9.40 ± 4.06 µg/kg). Higher Pb concentrations in fruit and vegetable products may come from contaminated soil or production processes [36]. The median of Pb in baby products from Brazil is significantly higher than in our research. It was shown that the median Pb was 33 μg/kg; therefore, the FAO/WHO standards were exceeded in 63% of Pb [28]. In the French study assessing the Pb content in food for infants and children, Pb was found in most of the analyzed samples (mean 2.4 µg/kg). The highest concentrations of Pb were found in the group of biscuits and bars (9.6 µg/kg), croissants (8.2 µg/kg), and cake and bread (5.5 µg/kg). Among all of the products, the highest content was found in chocolate biscuits (26.2 µg/kg), chocolate flakes for babies (19.7 µg/kg), and spinach (16.1 µg/kg). The products containing chocolate were characterized by significantly higher Pb concentrations [37]. In another Polish study (2012), the average consumption of desserts for children covers 2.2% of the PTDI of Pb, while the average consumption of a bottle of a drink for children is 13.6% of the PTDI of Pb [33]. In a Japanese study (2019), the weekly Pb exposure for children aged 1–3.5 years was 3.28 ± 0.26 μg/kg BW/week. Pb concentration was higher in children than in adults, but it did not exceed the acceptable standard [38]. In another study, the content of Pb was 1.27 μg/kg BW/day, which is 35.3% of the TDI. In our study, the EDI values for Pb are comparable (1.006 ± 1.192 μg/kg) [37]. In 18 samples, Pb was above the threshold values, and the highest amounts were found in drinks (7) and snacks (6). Pb contamination can come from fruits and grains, which are the main ingredient of these products. In the study by Kim et al, Pb was also present in high concentrations in fruits [27]. The source of Pb can be soil and water from highly industrialized areas. Fruit and grains are one of the main components of the diet of infants and young children, so they should not be abandoned, but it might be worthwhile to encourage children to be more varied in their diet to minimize the risk of possible Pb exposure. Exposure to this element disturbs the development of the central nervous system, and causes problems with concentration; thus, it is important to avoid chronic exposure.

Our results indicate that baby food may be safe, and the estimated health risk indicators did not show an increased health risk in terms of exposure of toxic elements in ready-made products for children. However, it is worrying that some products have exceeded the daily limit for Pb (18 samples) and Hg (6 samples). In Poland, around 60% of parents use convenience food, which should cause concern, especially in children, who often base their nutrition on ready-to-eat foods [39].

According to our results, it is worth paying attention to ready-to-eat products containing fish. In the study, children’s meals containing salmon had the highest levels of Cd (20.15 µg/kg), and those containing hake had the highest levels of Hg (37.25 µg/kg). Among the assortment of lunches intended for children over 1 year of age was a ready-made tuna dinner that should not be eaten at all in this age group. Some fish products are especially recommended for children (salmon, cod, mackerel, eel), while, due to the large accumulation of pollutants harmful to health, perch, shark, swordfish, and tuna are not recommended. The consumption of fish by children brings many health benefits, so we should not exclude them in the diet of children, while parents should pay attention to the selection of fish species that do not pose a health risk [40].

In our study, cereal products (especially those containing rice and rice products) contained high amounts of As (rice bar 84.71 µg/kg, baby porridge group, waffles and crips group). Cereals for children and snacks were characterized by one of the higher concentrations of toxic elements (in the case of As, gluten-free and gluten-free cereals; in the case of Cd, waffles/crips; in the case of Hg, porridges; in the case of Pb, snacks “for the hand”).

This allows us to conclude that cereal products easily accumulate toxic elements and, if consumed in large amounts, may pose a health hazard to infants and young children.

An unexpected observation is the high contribution of Cd in the group of beverages for children (4.17 ± 5.98 µg/kg), and the exceedance of the daily limit of Pb content in beverages (7 samples, 1.76% of all trials). This may result from contamination of the water used for the production of beverages or flavored waters. In Poland, there is an obligation to regularly test water for, e.g., toxic elements by provincial sanitary and epidemiological stations. This water has the correct parameters, while the problem may be an outdated sewage system located, for example, close to a factory, from which harmful substances, including toxic elements, may be released. Table 8 shows the content of toxic elements in baby food compared to the results of other authors.

## 5. Conclusions

The range of ready-to-eat products for children aged 0.5–3 years is a source of toxic elements, but most of the assessed products shown do not pose a health risk. In our study, the estimated health risk indicators did not show an increased health risk in terms of exposure to toxic elements in ready-made products for children. In some samples, Hg and Pb concentrations were exceeded, which may suggest that these products are not completely safe for children. There is a need for monitoring in ready-to-eat products intended for children aged 0.5–3 years. Particular attention should be paid to children’s consumption of fish-based products, cereal products, snacks, and flavored drinks and waters. These product groups should be subject to special control with regard to the health safety of children already at the production stage.

## Figures and Tables

**Table 1:** The number of samples with product categories.

Type of Products	*n*
BABY DINNERS	102
-with poultry	24
-with beef	16
-with pork	13
-with fish	18
-with rabbit	11
-vegetarian	20
PORRIDGE	50
-with milk	8
-with milk and fruit	15
-cereal gluten	12
-gluten-free cereal	15
FRUIT AND VEGETABLE MOUSSES	58
-fruit and vegetables	9
-fruit	33
-fruit and cereal	6
-fruit and dairy	6
-vegetables	4
BABY DRINKS	64
-fruit drinks and water	22
-fruit juices	42
SNACKS “FOR THE HAND”	62
-waffle/crisps	30
-biscuits/cookies	17
-fruit bars	15
DAIRY	60
-yellow cheese	28
-yogurt	32
TOTAL	397

**Table 2:** The results of the quality control of the certified reference materials.

Element	Declared Concentration in CRM (µg/kg)	Recovery (%)	Precision (%)
CORN FLOUR (INCT-CF-3)			
As	10 *	101.3	4.5
Cd	7 *	99.1	4.1
Hg	1.5 *	101.7	4.6
Pb	52 *	98.8	2.3
SKIM MILK POWDER (CRM 63R)			
Hg	0.19 ± 0.02	98.3	3.8
Pb	18.5 ± 2.7	100.9	4.3
SIMULATED DIET D			
As	<50 *	-	3.9
Cd	478 ± 26	98.4	2.4
Hg	52 *	102.0	3.2
Pb	218 ± 13	99.6	2.2

* Informational value.

**Table 3:** Maximum allowable content of As, Cd, Hg, and Pb in food and the number of baby food samples that exceed the maximum allowable content [16,17,22].

Baby Food Samples	Maximum Allowable Content (mg/kg Product Weight)				Exceeding Maximum Allowable Content							
					As		Cd		Hg		Pb	
	As	Cd	Hg	Pb	*n*	%	*n*	%	*n*	%	*n*	%
Dinners (*n* = 102)	-	0.04	0.01	0.02	-	-	0	0	3	0.75	1	0.25
Porridges (*n* = 50)	0.5	0.04	0.01	0.02	0	0	0	0	0	0.00	2	0.50
Mousses (*n* = 58)	0.5	0.02	0.01	0.01	0	0	0	0	2	0.50	1	0.25
Drinks (*n* = 64)	-	0.02	0.02	0.02	-	-	0	0	1	0.25	7	1.76
Baby snacks (*n* = 62)	-	0.04	0.01	0.02	-	-	0	0	0	0.00	6	1.50
Dairy (*n* = 60)	-	-	0.01	0.02	-	-	-	-	0	0.00	1	0.25
TOTAL (*n* = 397)					-	-	-	-	6	1.50	18	4.53

*n*—number of samples, %—percentage of exceeded samples.

**Table 4:** Significant statistical differences in the analyzed product groups.

Toxic Elements	Analyzed Food Group	*p*-Value
As	Dinners—drinks	<0.01
	Porridges—drinks	<0.01
	Mousses—drinks	<0.01
	Baby snacks—drinks	<0.01
	Dairy—drinks	<0.01
	Poultry dinners—fish dinners	<0.001
	Beef dinners—fish dinners	<0.001
	Pork dinners—fish dinners	<0.001
	Rabbit dinners—fish dinners	<0.001
	Vegetarian dinners—fish dinners	<0.001
	Milk porridge—gluten-free cereal	<0.001
Hg	Mousses—dairy	<0.05
	Pork dinners—vegetarian dinners	<0.05
Pb	Fruit drinks and water—fruit juices	<0.01

**Table 5:** Average value of the ratios: EDI, EWI, PTWI, and BMDL in all analyzed groups.

Type of Products	Elements	EDI (µg/Day)	EWI (µg/Week)	PTWI (%PTWI) (µg/kg/BW/Week)	BMDL (%BMDL) (µg/kg/BW/Day)
Baby dinners (*n* = 102)	As	0.419 ± 0.909	2.938 ± 6.368	NA	0.042 ± 0.091 (1.40%)
	Cd	0.515 ± 0.744	3.607 ± 5.205	0.362 ± 0.523 (5.17%)	NA
	Hg	0.544 ± 0.579	3.804 ± 4.049	0.382 ± 0.407 (23.89%)	NA
	Pb	1.651 ± 0.922	11.563 ± 6.458	NA	0.166 ± 0.092 (33.2%)
Porridges (*n* = 50)	As	0.051 ± 0.054	0.362 ± 0.380	NA	0.005 ± 0.005 (0.17%)
	Cd	0.064 ± 0.065	0.449 ± 0.459	0.045 ± 0.046 (0.64%)	NA
	Hg	0.097 ± 0.057	0.681 ± 0.402	0.068 ± 0.040 (4.28%)	NA
	Pb	0.187 ± 0.114	1.313 ± 0.804	NA	0.018 ± 0.011 (3.77%)
Mousses (*n* = 58)	As	0.021 ± 0.017	0.148 ± 0.119	NA	0.002 ± 0.001 (0.07%)
	Cd	0.159 ± 0.226	1.0113 ± 1.583	0.111 ± 0.159 (1.59%)	NA
	Hg	0.381 ± 0.239	2.669 ± 1.677	0.268 ± 0.168 (16.76%)	NA
	Pb	0.892 ± 2.008	6.245 ± 14.056	NA	0.08 ± 0.02 (17.93%)
Baby drinks (*n* = 64)	As	0.037 ± 0.086	0.264 ± 0.607	NA	0.003 ± 0.008 (0.12%)
	Cd	0.078 ± 0.165	0.548 ± 1.551	0.055 ± 0.116 (0.78%)	NA
	Hg	0.122 ± 0.080	0.858 ± 0.560	0.086 ± 0.056 (5.39%)	NA
	Pb	0.553 ± 0.320	3.874 ± 2.242	NA	0.055 ± 0.032 (11.12%)
Snacks (*n* = 62)	As	0.242 ± 0.870	1.697 ± 6.091	NA	0.024 ± 0.087 (0.81%)
	Cd	0.252 ± 0.226	1.767 ± 1.585	0.177 ± 0.159 (2.53%)	NA
	Hg	0.192 ± 0.136	1.348 ± 0.958	0.135 ± 0.096 (8.46%)	NA
	Pb	1.053 ± 0.610	7.375 ± 4.276	NA	0.105 ± 0.061 (21.17%)
Dairy (*n* = 60)	As	0.007 ± 0.034	0.054 ± 0.233	NA	0.000 ± 0.003 (0.02%)
	Cd	0.063 ± 0.081	0.436 ± 0.563	0.044 ± 0.056 (0.63%)	NA
	Hg	0.391 ± 0.668	2.726 ± 4.641	0.275 ± 0.470 (17.21%)	NA
	Pb	1.168 ± 1.491	8.047 ± 10.397	NA	0.117 ± 0.149 (23.47%)
TOTAL (*n* = 397)	As	0.162 ± 0.598	1.139 ± 4.196	NA	0.016 ± 0.060 (0.54%)
	Cd	0.224 ± 0.439	1.571 ± 3.076	0.157 ± 0.309 (2.25%)	NA
	Hg	0.314 ± 0.438	2.204 ± 3.070	0.221 ± 0.308 (13.84%)	NA
	Pb	1.006 ± 1.192	7.046 ± 8.348	NA	0.101 ± 0.119 (20.23%)

BMDL—the benchmark dose lower confidence limit, EDI—estimated daily intake, EWI—estimated weekly intake, NA—not applicable, PTMI—provisional tolerable monthly intake, PTWI—provisional tolerable weekly intake.

**Table 6:** Estimated values of THQ and CR indices of toxic elements (As, Cd, Hg, and Pb) in the analyzed food products.

THQ X ± SD (Min–Max)				
Type of Products	As	Cd	Hg	Pb
Baby dinners (*n* = 102)	1.41 × 10^−7^ ± 3.04 × 10^−7^ (9.61 × 10^−7^–1.26 × 10^−6^)	2.26 × 10^−8^ ± 4.41 × 10^−8^ (1.75 × 10^−9^–1.56 × 10^−6^)	1.01 × 10^−7^ ± 1.19 × 10^−7^ (1.36 × 10^−10^–8.56 × 10^−7^)	1.06 × 10^−7^ ± 1.46 × 10^−7^ (1.75 × 10^−9^–1.56 × 10^−6^)
Porridges (*n* = 50)	1.82 × 10^−8^ ± 1.82 × 10^–8^ (1.23 × 10^−8^–2.44 × 10^−6^)	1.31 × 10^−8^ ± 2.01 × 10^−8^ (1.82 × 10^−8^–2.31 × 10^−6^)	1.06 × 10^−7^ ± 1.46 × 10^−7^ (7.66 × 10^−10^–8.65 × 10^−7^)	1.01 × 10^−7^ ± 1.19 × 10^−7^ (1.88 × 10^−8^–8.65 × 10^−7^)
Mousses (*n* = 58)	7.11 × 10^−9^ ± 5.70 × 10^−9^ (1.31 × 10^−8^–2.79 × 10^−6^)	2.13 × 10^−8^± 2.44 × 10^−8^ (1.14 × 10^−11^–8.78 × 10^−7^)	1.06 × 10^−7^ ± 1.19 × 10^−7^ (1.36 × 10^−10^–8.65 × 10^−7^)	c3.21 × 10^−7^ ± 2.88 × 10^−7^ (7.03 × 10^−9^–8.55 × 10^−6^)
Baby drinks (*n* = 64)	1.29 × 10^−8^± 2.92 × 10^−8^ (1.31 × 10^−8^–1.47 × 10^−6^)	3.06 × 10^−8^ ± 4.40 × 10^−8^ (8.54 × 10^−11^–4.46 × 10^−7^)	1.98 × 10^−7^ ± 1.70 × 10^−7^ (2.06 × 10^−10^–4.50 × 10^−6^)	2.98 × 10^−7^ ± 3.17 × 10^−7^ (1.05 × 10^−9^ –1.26 × 10^−6^)
Snacks (*n* = 62)	8.12 × 10^−8^ ± 2.91 × 10^−7^ (5.95 × 10^−9^–2.31 × 10^−6^)	4.99 × 10^−8^ ± 1.10 × 10^−8^ (8.52 × 10^−11^–9.89 × 10^−7^)	1.76 × 10^−7^ ± 4.46 × 10^−7^ (6.11 × 10^−11^–1.05 × 10^−7^)	4.51 × 10^−7^ ± 1.19 × 10^−7^ (1.46 × 10^−10^–1.16 × 10^−6^)
Dairy (*n* = 60)	2.66 × 10^−9^ ± 1.127 × 10^−8^ (28.84 × 10^−10^–2.01 × 10^−7^)	2.63 × 10^−9^ ± 2.33 × 10^−8^ (1.47 × 10^−11^–1.46 × 10^−7^)	1.14 × 10^−7^ ± 6.18 × 10^−7^ (1.99 × 10^−10^–8.21 × 10^−7^)	1.91 × 10^−7^ ± 1.19 × 10^−7^ (1.75 × 10^−9^–1.56 × 10^−7^)
TOTAL (*n* = 397)	5.44 × 10^−8^ ± 2.01 × 10^−8^ (28.84 × 10^−10^–1.26 × 10^−6^)	2.61 × 10^−8^ ± 4.42 × 10^−8^ (1.47 × 10^−11^–1.56 × 10^−6^)	1.65 × 10^−7^ ± 1.47 × 10^−7^ (6.11 × 10^−11^–4.50 × 10^−6^)	2.82 × 10^−7^ ± 2.21 × 10^−7^ (1.46 × 10^−10^–1.16 × 10^−6^)
Mean CR	2.44 × 10^−7^ ± 8.97 × 10^−7^ (2.07 × 10^−7^–1.03 × 10^−5^)	1.41 × 10^−6^ ± 2.77 × 10^−6^ (5.34 × 10^−6^–2.79 × 10^−5^)	NA	8.56 × 10^−9^ ± 1.01 × 10^−8^ (1.48 × 10^−10^–1.32 × 10^−7^)

CR—cancer risk, Max—maximum, Min—minimum, NA—not applicable, SD—standard deviation, THQ—target hazard quotient, X—mean.

**Table 7:** The average content and median of toxic elements in the studied groups and significant statistical differences, taking into account the intended use of products for age groups.

Average Content of Analyzed Parameters							Statistical Differences		
Type of Products	Elements	*n*	X ± SD (µg/kg)	Min–Max (µg/kg)	Me (µg/kg)	Q1–Q3 (µg/kg)	Toxic Elements	Analyzed Food Group	*p*-Value
for children 6–12 months (a)	As Cd Hg Pb	107	2.01 ± 3.67 2.22 ± 3.14 3.20 ± 2.86 7.82 ± 4.76	0.00–16.97 0.31–20.15 0.01–9.88 0.52–32.28	0.44 1.33 2.05 6.70	0.17–1.72 0.68–2.02 0.83–5.28 5.25–9.89	As	a/b b/c	<0.01 <0.001
for children under 12 months (b)	As Cd Hg Pb	104	1.01 ± 2.54 2.17 ± 2.78 3.09 ± 2.22 7.74 ± 13.55	0.00–14.63 0.16–13.34 0.17–9.12 0.77–134.00	0.19 1.15 2.53 5.81	0.16–0.50 0.64–2.20 1.240–4.47 4.72–7.45	Cd	a/c b/c	<0.001 <0.01
without an age declaration (c)	As Cd Hg Pb	186	1.35 ± 6.42 1.99 ± 3.23 2.68 ± 1.86 10.95 ± 7.02	0.00–84.71 0.14–18.54 0.00–9.12 0.460–48.18	0.20 0.63 2.11 9.38	0.00–0.66 0.27–2.24 1.27–3.74 7.18–12.09	Pb	a/b a/c b/c	<0.05 <0.001 <0.001

a—products for children 6–12 months, b—products for children under 12 months, c—products without an age declaration, Max—maximum, Me—median, Min—minimum, SD—standard deviation, Q1—quartile 1, Q3—quartile 3, X—mean.

**Table 8:** The content of toxic elements in baby food shown in studies by other authors.

Type of Products	As (μg/kg)	Cd (μg/kg)	Hg (μg/kg)	Pb (μg/kg)	Reference
Baby food *	NA	Me = 2.8 Max = 103.9	NA	Max = 183.6	[15]
Baby desserts	NA	Me = 0.71 Max = 14.90	NA	Me = 2.14	[30]
Baby juices	NA	Me = 0.80 Max = 1.34	NA	Max = 5.11	
Baby dinners	NA	Me = 8.31 Max = 28.2	NA	Me = 20.6 Max = 41.0	
Baby food	<LOD	<LOD	NA	<LOD	[23]
	Max = 0.89	Max < LOD	NA	<LOD	
Baby dinners	NA	NA	Me = 0.80 Max = 7.40	NA	[33]
Cereals food	NA	NA	Me = 0.58 Max = 1.0	NA	
Rise baby food	Me = 0.088	NA	Me = 0.062	NA	[24]
	Max = 0.150	NA	Max = 0.3	NA	
Rise baby food	NA	NA	X = 0.94 ± 0.47	NA	[14]
Milk-based food	X = 0.23 ± 0.05	X = 0.23 ± 0.05	NA	X = 1.2 ± 0.19	[25]
Spelt based food	X = 3.8 ± 0.05	X = 2.4 ± 0.11	NA	X = 1.8 ± 0.12	
Oat-based food	X = 2.4 ± 0.19	X = 3.3 ± 0.14	NA	X = 3.1 ± 0.23	
Rice-based food	X = 1.7 ± 0.04	X = 33 ± 0.56	NA	X = 1.2 ± 0.12	
Baby food	NA	X = 0.38 ± 0.2	X = 0.22 ± 0.08	X = 0.47 ± 0.14	[27]
Cereal-based food	NA	NA	Me = 0.50	NA	[34]
Baby food	NA	NA	Me = 0.40	NA	
Baby food	NA	Me = 33.0	NA	Me = 109	[28]
Milk-based food	NA	X = 0.05 ± 0.005	NA	X = 0.21 ± 0.02	[32]
Milk-based food	NA	NA	Me = 0.03	NA	[41]
Fruit-based food	<LOD	<LOD	NA	X = 12.0	[36]
Vegetable-based food				X = 29.0	
Meat based food				X = 34.50	
Cereal-based food	X = 0.68 ± 0.67	NA	NA	NA	[26]
Baby food	NA	NA	NA	X = 3.4 ± 2.01	[37]
Milk-based food				X = 1.11 ± 0.21	
Cereal-based food				X = 1.40 ± 1.95	
Baby mouses				X = 2.15 ± 2.08	
Baby drinks				X = 3.72 ± 2.31	

* Baby food—vegetable, meat, fish, fruit-based samples, LOD—limits of detection, Max—maximum, Me—median, Min—minimum, NA—not applicable, X—mean.

## Data Availability

Detailed data are available from the authors.

## Supplementary Materials

The following supporting information can be downloaded at: https://www.mdpi.com/article/10.3390/nu14112325/s1, Table S1: The content of toxic elements in tested products.
