# Supplementary material for: Health Safety Assessment of Ready-to-Eat Products Consumed by Children Aged 0.5–3 Years on the Polish Market
Source: Nutrients. 2022 Jun 1;14(11):2325. doi: 10.3390/nu14112325 (PMC9183086; doi:10.3390/nu14112325)
Supplement: Supplementary file 1 [file nutrients-14-02325-s001.zip › nutrients-1735745-supplementary.pdf]

nutrients

MDPI

Supplementary Materials

Health Safety Assessment of Ready-to-Eat Products Consumed by Children Aged 0.5–3 Years on the Polish Market

Anita Żmudzińska *, Anna Puścion-Jakubik, Joanna Bielecka, Monika Grabia, Jolanta Soroczyńska, Konrad Mielcarek and Katarzyna Socha

Department of Bromatology, Faculty of Pharmacy with the Division of Laboratory Medicine, Medical University of Białystok, Mickiewicza 2D Street, 15-222 Białystok, Poland; anna.puscion-jakubik@umb.edu.pl (A.P.-J.); joanna.bielecka@umb.edu.pl (J.B.); monika.grabia@umb.edu.pl (M.G.); jolanta.soroczynska@umb.edu.pl (J.S.); konrad.mielcarek@umb.edu.pl (K.M.); katarzyna.socha@umb.edu.pl (K.S.)

* Correspondence: anita.zmudzinska@umb.edu.pl; Tel.: +48-85-748-5469

Table S1. The content of toxic elements in tested products.

Type of products	*n*	As (µg/kg)	Cd (µg/kg)	Hg (µg/kg)	Pb (µg/kg)
		X ± SD (Min-Max) Me(Q1-Q3)	X ± SD (Min-Max) Me(Q1-Q3)	X ± SD (Min-Max) Me(Q1-Q3)	X ± SD (Min-Max) Me(Q1-Q3)
BABY DINNERS	102	1.86 ± 0.42 (<LOD-16.97) 0.19(0.16-0.67)	2.31 ± 0.33 (0.30-20.15) 1.22(0.78-2.03)	2.97 ± 0.44 (<LOD-37.25) 1.46(0.51-4.02)	7.55 ± 0.41 (0.52-32.27) 6.76(5.57-9.17)
-with poultry	24	0.24 ± 0.04 (<LOD -0.79) 0.17(0.15-0.32)	1.19 ± 0.14 (0.42-2.72) 1.07(0.62-1.79)	3.54 ± 1.55 (<LOD-37.25) 0.97(0.34-3.58)	8.46 ± 1.29 (0.76-32.27) 7.32(5.65-10.43)
-with beef	16	0.34 ± 0.06 (0.15-1.17) 0.24(0.16-0.42)	1.77 ± 0.41 (0.51-7.35) 1.25(0.73-2.06)	2.50 ± 3.36 (0.23-10.26) 0.64(0.52-4.24)	5.32 ± 0.76 (0.52-10.81) 5.38(3.33-6.36)
-with pork	13	0.28 ± 0.058 (<LOD-0.69) 0.26(0.16-0.33)	3.31 ± 1.08 (0.57-11.79) 1.73(0.57-11.79)	1.64 ± 0.74 (<LOD-9.51) 0.37(0.16-2.03)	7.24 ± 1.12 (2.26-17.13) 6.37(4.75-10.21)
-with fish	18	2.88 ± 1.27 (0.52-16.97) 1.94(1.11-3.48)	3.67 ± 1.35 (0.42-20.15) 1.38(5.90-9.17)	9.21 ± 0.57 (1.81-16.97) 10.52(3.60-13.04)	8.02 ± 0.63 (5.28-13.60) 7.06(5.90-9.17)
-with rabbit	11	0.31 ± 0.1 (<LOD-1.02) 0.18(0.16-0.36)	1.39 ± 0.27 (0.30-0.58) 1.14(0.58-1.87)	2.59 ± 0.96 (<LOD-9.87) 1.23(0.20-4.51)	7.87 ± 1.27 (1.86-15.63) 7.82(5.40-10.46)
-vegetarian	20	0.29 ± 0.06 (0.13-1.11) 0.161(0.15-0.31)	2.74 ±0.77 (0.31-11.56) 1.63(0.96-2.07)	3.86 ± 0.68 (1.08-11.83) 2.44(1.83-5.11)	7.86 ± 0.52 (5.47-13.03) 1.63(0.96-2.07)
PORRIDGE	50	2.30 ± 0.33 (<LOD-8.48) 1.11(0.62-3.60)	2.77 ± 0.40 (0.46-16.03) 1.71(1.25-3.12)	4.2 ± 0.35 (0.72-9.11) 4.20(1.41-6.04)	8.09 ± 0.70 (1.96-25.37) 6.28(5.34-8.26)
-with milk	8	0.61 ± 0.12 (<LOD-1.16) 0.63 (0.46-0.76)	1.66 ± 0.49 (0.68-5.03) 1.31(1.00-1.45)	3.01 ± 1.06 (0.95-8.97) 1.28(1.03-4.76)	6.21 ± 0.82 (4.42-10.38) 5.16(4.76-7.52)

-with milk and fruit	15	1.47 ± 0.32<br>(0.23-4.40)<br>0.95(0.58-2.24)	1.98 ± 0.33<br>(0.46-4.68)<br>1.48(1.10-2.92)	3.60 ± 0.54<br>(0.72-6.35)<br>3.45(1.41-6.04)	7.90 ± 1.4<br>(3.97-24.26)<br>6.18(5.1-7.14)
-cereal gluten	12	1.66 ± 0.54<br>(0.21-6.95)<br>0.97(0.21-6.95)	3.30 ± 0.83<br>(0.67-9.31)<br>2.07(1.28-4.70)	4.86 ± 0.59<br>(1.25-8.99)<br>5.04(3.39-5.75)	8.61 ± 1.58<br>(1.96-17.93)<br>6.95(4.84-12.07)
-cereal gluten free	15	4.31 ± 0.72<br>(0.18-8.48)<br>4.47(1.55-7.40)	3.72 ± 1.04<br>(0.69-16.03)<br>2.09(1.41-4.25)	4.89 ± 0.69<br>(0.91-9.11)<br>6.00(1.52-6.33)	8.87 ± 1.36<br>(5.35-25.37)<br>6.75(5.63-9.52)
FRUIT AND VEGETABLE MOUSSES	58	0.19 ± 0.48<br>(<LOD-0.54)<br>0.17(0.147-0.311)	1.39 ± 2.32<br>(0.15-10.14)<br>0.66(0.49-1.46)	3.93± 0.26<br>(0.95-23.86)<br>3.12(1.73-5.01)	7.97± 0.02<br>(3.03-138.99)<br>4.95(4.41-6.61)
-fruit and vegetables	9	0.21 ± 2.43<br>(<LOD-0.54)<br>0.17(0.14-0.34)	2.27 ± 0.88<br>(0.37-9.77)<br>0.65(0.56-1.23)	5.75 ± 1.12<br>(1.01-23.86)<br>3.66(1.24-5.45)	5.98 ± 0.06<br>(3.95-10.60)<br>0.17(0.14-0.34)
-fruit	33	0.19 ± 0.03<br>(<LOD-0.40)<br>0.17 (<LOD-0.31)	1.22 ± 0.33<br>(0.16-10.14)<br>0.65 (0.41-0.90)	3.77 ± 0.46<br>(0.95-13.24)<br>3.24(2.10-5.01)	9.40 ± 4.06<br>(3.03-138.9)<br>4.84(0.00-0.31)
-fruit and cereal	6	0.16 ± 0.09<br>(<LOD-0.29)<br>0.17(0.15-0.17)	0.74 ± 0.54<br>(0.15-1.56)<br>0.57(0.39-1.22)	3.47 ± 2.50<br>(1.72-8.21)<br>2.42(1.76-4.27)	6.58 ± 4.27<br>(4.00-15.19)<br>5.00(4.46-5.80)
-fruit and dairy	6	0.23 ± 0.14<br>(<LOD-0.36)<br>0.25(0.17-0.35)	1.24 ± 2.30<br>(0.24-3.33)<br>0.72(0.42-2.01)	3.71 ± 2.30<br>(1.07-6.35)<br>3.44(1.72-6.21)	6.24 ± 1.47<br>(3.92-7.69)<br>6.47(5.28-7.58)
-vegetables	4	0.18 ± 0.15<br>(<LOD-0.37)<br>0.18(0.08-0.28)	1.93 ± 0.27<br>(1.68-2.19)<br>1.92(1.69-2.16)	2.26 ± 0.83<br>(1.55-3.27)<br>2.11(1.58-2.93)	5.41 ± 1.91<br>(3.54-8.05)<br>5.01(4.13-6.67)
BABY DRINKS	64	0.88 ± 2.04<br>(<LOD-9.09)<br>0.15(0.14-0.29)	3.39 ± 1.3<br>(0.14-18.54)<br>0.48(0.29-1.14)	2.89 ± 1.87<br>(1.00-9.11)<br>2.31(1.45-3.78)	1.14 ± 0.98<br>(0.46-3.96)<br>9.98(8.88-13.33)
-fruit drinks and water	22	2.27 ± 3.13<br>(<LOD-9.09)<br>0.16(0.13-3.73)	4.17 ± 5.98<br>(0.16-18.54)<br>1.2(0.50-5.02)	2.90 ± 1.62<br>(1.00-7.38)<br>1.01(1.87-3.73)	1.79 ± 1.97<br>(0.46-3.96)<br>1.42(0.46-3.97)
-fruit juices	42	0.19 ± 0.15<br>(<LOD-0.65)<br>0.15(0.14-0.27)	0.66 ± 2.01<br>(0.14-6.82)<br>0.38(0.28-0.58)	2.88 ± 2.00<br>(1.00-9.11)<br>2.1(1.41-4.09)	1.03 ± 0.27<br>(0.57-2.16)<br>0.98(0.87-1.10)
SNACKS “FOR THE HAND”	62	2.92 ± 10.77<br>(0.19-2.91)<br>0.78(0.38-2.00)	3.09 ± 1.69<br>(0.46-16.08)<br>2.29(1.43-3.44)	2.36 ±1.69 (<LOD-7.02)<br>1.97(1.05-3.25)	12.8 ± 7.56 (4.54-48.18)<br>10.68(8.85-14.30)
-waffle/crisps	30	4.88 ± 15.34<br>(0.19-84.71)<br>0.78(0.38-3.26)	3.51 ± 3.38<br>(0.46-16.08)<br>2.62(1.31-3.80)	2.23 ± 1.59<br>(0.34-6.46)<br>1.46(1.2-3.2)	13.83 ± 9.08<br>(4.54-48.18)<br>11.83(9.22-15.06)
-biscuits/cookies	17	0.93 ± 0.82<br>(0.20-2.72)<br>0.71(0.22-1.22)	2.58 ± 1.30<br>(1.19-6.68)<br>2.43(1.63-2.99)	2.33 ± 2.10<br>(<LOD-7.02)<br>1.91(1.03-2.70)	10.4 ± 4.40<br>(7.01-23.72)<br>9.17(8.01-10.81)
-fruit bars	15	1.27 ± 0.86<br>(0.21-2.91)<br>1.15(0.47-1.66)	2.83 ± 2.82<br>(0.62-9.81)<br>1.83(0.78-2.85)	2.65 ± 1.47<br>(0.73-5.36)<br>2.32(1.31-4.01)	14.15 ± 6.73<br>(7.75-30.99)<br>12.09(9.43-14.56)
DAIRY	60	0.10 ± 0.16<br>(<LOD-0.80)	0.95 ± 2.56<br>(0.11-17.79)	3.02 ± 2.56<br>(0.11-15.23)	6.66 ± 3.11 (2.13-21.01)

		<LOD(<LOD-0.80)	2.13(1.35-4.50)	2.13(1.35-4.50)	6.11(4.71-7.29)
-yellow cheese	28	0.11 ± 0.15<br>(<LOD-0.58)<br><LOD(<LOD-0.19)	1.51 ± 3.56<br>(0.11-17.79)<br>0.25(0.20-0.66)	2.94 ± 2.99<br>(1.00-15.23)<br>1.96(1.40-2.85)	7.5 ± 3.90<br>(3.54-21.01)<br>6.51(5.08-8.59)
-yogurt	32	0.1 ± 0.17<br>(<LOD-0.80)<br><LOD(<LOD-0.181)	0.45 ± 0.78<br>(0.19-4.64)<br>0.26(0.21-0.40)	3.09 ± 2.16<br>(0.11-7.87)<br>2.73(1.31-4.83)	5.93 ± 2.0<br>(2.13-11.46)<br>6.04(4.54-6.92)
TOTAL	397	1.41 ± 0.25<br>(<LOD-84.71)<br>0.23(0.15-0.77)	2.08 ± 0.15<br>(0.11-20.15)<br>1.12(0.49-2.08)	3.16 ± 0.16<br>(<LOD-37.25)<br>2.11(1.21-4.36)	9.27 ± 0.44<br>(0.46-138.99)<br>7.64(5.46-10.31)

LOD – limits of detection, max – maximum , min – minimum, SD – standard deviation, X – mean.
